# News and Coming Events

**Published:** 1907-04-13

**Authors:** 


### NEWS AND COMING EVENTS.

The new isolation hospital at Merthyr, completed at a cost of £17,000, has just been opened.

The Swindon Victoria Hospital has received the sum of £700, anonymously, for the purpose of endowing a bed.

Her Royal Highness Princess Louise, Duchess of Argyle, has graciously consented to open the new out-patient hall of the London Temperance Hospital, on Tuesday, May 14 next.

Dr. Robert R. Rentoul, of Liverpool, will speak on "Woman's Health: Our Greatest National Asset," at 34 Devonshire Street, Harley Street, W., at 3.30 p.m. on Monday, April 15.

The Royal Dental Hospital, Leicester Square, has received a legacy of £1,000 for the general purposes of the hospital, also £1,000 for the endowment of a scholarship in dental surgery, under the will of the late Mr. Alfred James Woodhouse.

On Saturday last the Cordingley Motor Show was opened in the Agricultural Hall, Islington, N. A large number of cars and motor appliances, many of them of special interest to medical men, are on show. The exhibition will remain open until Saturday, April 13.

At the meeting of the Therapeutical Society to be held next Tuesday, April 16, in the Apothecaries' Hall, Blackfriars, E.C., at 4.30 p.m., Dr. George Oliver will read a paper on "Some Notes on the Control of Hypernormal Blood-pressure," and Dr. James Burnet one on "The Decline of Prescription Writing: Some of its Dangers and Ultimate Results."

At the annual meeting of the Hospital Saturday Fund on Saturday last, the Council reported a continuance of the steady progress made by the Fund since the abolition of the street collection. The income for the year was £26,460, an increase of £530; and £23,898 has been distributed among 205 institutions. Some 41,686 letters of recommendation has been distributed; the Surgical Appliance Committee has provided 5,321 appliances at a cost of £4,372 (towards which, it is satisfactory to notice, the patients had contributed £3,114). The report was adopted. The Lord Mayor was elected President of the Fund.

The annual meeting of the Home Hospitals Association, under the presidency of Mr. J. Bland Sutton, F.R.C.S., was held on March 26. The chairman, in moving the adoption of the report, said that the finances of the Association were in a thoroughly sound condition, and, largely owing to the generosity of the Duke of Northumberland and the Treasurer, Mr. Cox, Fitzroy House could claim to be the best-equipped private nursing home in London. He pointed out that at Fitzroy House a patient could select his own room according to his pocket or inclination, and that extras were few. The nurses were fully trained and had been specially selected, and the institution was not a training home for nurses. The retiring President, the Duke of Northumberland; the retiring Treasurer, Mr. Cox; the auditors, Messrs. Chatteris, Nichols and Co.; and Sir John Watney and Mr. Frederick Cox, as members of the Committee, were re-elected. Votes of thanks to the nursing staff and to Mr. Sutton for presiding concluded the meeting.

Mr. R. Glynn Vivian, of the firm of Vivian and Sons, has given £3,600 for the establishment of a convalescent home for the blind at Caswell Bay.

Dr. C. Saleeby will open the discussion on "Alcohol and Civilisation" at the next meeting of the Society for the Study of Inebriety, to be held at 11 Chandos Street, Cavendish Square, on Tuesday, July 9.

The executors of the late Mr. John Lawrence Toole have made a grant of £500, less duty, to the Royal Hospital for Incurables, Putney Heath, an institution to which the deceased was for many years a regular subscriber, and in the work of which he took a deep personal interest.

"Anon" has given £500 to the festival dinner list of Alderman Sir Vaughan Morgan, Bart., in the hope that five others will follow his example. The dinner is in aid of the needy funds of the Royal Hospital for Incurables, Putney Heath, and will be held at the Whitehall Rooms on May 16.

The committee of the International Congress of Hygiene, to be held in Berlin on September 23 next, has made arrangements to afford opportunities to the members of the Congress of visiting the numerous hygienic institutions. Over one hundred establishments will be thrown open for inspection, and it is intended to publish a special guide-book in three languages descriptive of these institutions. The office of Dr. Nietner, the Secretary General, is at 9 Eichhornstr, Berlin, W.

Mr. Michelli, Secretary of the Seamen's Hospital, Greenwich, in a letter to the *Times*, draws attention to the Employers' Liability Act which comes into force on July 1, and to the effect which it will produce upon voluntary hospitals. "Under the new Act," writes Mr. Michelli, "the assistance rendered by these institutions will be materially extended, and the voluntary hospitals will be daily employed in saving many thousands of pounds for the employers of labour and the insurance companies at the expense of the charitable public." He points out that "it is only fair play for the insurance company to pay towards the maintenance in hospital of men to whom compensation is due; but there is no provision to this effect in the Act. When a man is doctored and nursed in his own home the compensation goes to pay the doctor and the nurse; then why not when he is doctored and nursed in a hospital?"

On Friday, April 12, the French Congress of General Practitioners will meet in Paris to discuss the burning question of reform in the existing system of medical teaching. There is a very general opinion that the system of examinations has been carried to excess, with the result that while students are offered every encouragement to study the theory of their art, practical work takes a secondary place. Diplomas won by success in the examination room are doubtless valuable as showing theoretical knowledge, but as a writer in *Le Journal* remarks, "ten years' school work cannot take the place of ten months of hospital work." The need for reform in this matter has long been recognised, and the referendum recently organised by Dr. Huchard shows that there is an overwhelming mass of professional opinion in favour of limiting the time devoted to preparation for examination, and giving more attention to practical work in the hospitals.

